# E3 Ubiquitin Ligase-Mediated Regulation of Osteoblast Differentiation and Bone Formation

**DOI:** 10.3389/fcell.2021.706395

**Published:** 2021-08-27

**Authors:** Jianlin Shen, Bowen Fu, Yanfang Li, Yanjiao Wu, Hongxun Sang, Heshi Zhang, Haibin Lin, Huan Liu, Wenhua Huang

**Affiliations:** ^1^Guangdong Innovation Platform for Translation of 3D Printing Application, Center for Orthopaedic Surgery, The Third Affiliated Hospital of Southern Medical University, Guangzhou, China; ^2^Department of Orthopedics, Affiliated Hospital of Putian University, Putian, China; ^3^Department of Pediatric Surgery, Affiliated Hospital of Putian University, Putian, China; ^4^Department of Orthopedics, Shunde Hospital of Southern Medical University, Guangzhou, China; ^5^Department of Orthopedics, Shenzhen Hospital, Southern Medical University, Shenzhen, China; ^6^Department of Vessel and Breast, Affiliated Traditional Chinese Medicine Hospital of Southwest Medical University, Luzhou, China; ^7^Department of Orthopedics, Affiliated Traditional Chinese Medicine Hospital, Southwest Medical University, Luzhou, China; ^8^Guangdong Engineering Research Center for Translation of Medical 3D Printing Application, Guangdong Provincial Key Laboratory of Medical Biomechanics, School of Basic Medical Sciences, Southern Medical University, Guangzhou, China; ^9^Orthopedic Center, Affiliated Hospital of Guangdong Medical University, Guangdong Medical University, Zhanjiang, China

**Keywords:** E3 ubiquitin ligase, transcription factor, signaling pathway, osteoblast differentiation, bone formation, therapeutic target

## Abstract

The ubiquitin–proteasome system (UPS) is an essential pathway that regulates the homeostasis and function of intracellular proteins and is a crucial protein-degradation system in osteoblast differentiation and bone formation. Abnormal regulation of ubiquitination leads to osteoblast differentiation disorders, interfering with bone formation and ultimately leading to osteoporosis. E3 ubiquitin ligases (E3) promote addition of a ubiquitin moiety to substrate proteins, specifically recognizing the substrate and modulating tyrosine kinase receptors, signaling proteins, and transcription factors involved in the regulation of osteoblast proliferation, differentiation, survival, and bone formation. In this review, we summarize current progress in the understanding of the function and regulatory effects of E3 ligases on the transcription factors and signaling pathways that regulate osteoblast differentiation and bone formation. A deep understanding of E3 ligase-mediated regulation of osteoblast differentiation provides a scientific rationale for the discovery and development of novel E3-targeting therapeutic strategies for osteoporosis.

## Introduction

Posttranslational modification mediated by the ubiquitin–proteasome system (UPS) plays a very important role in protein localization, metabolism, regulation, and degradation and is an essential process for maintaining protein homeostasis (Thibaudeau and Smith, 2019). Protein ubiquitination is a three-step process that involves ubiquitin activation by the E1 enzyme, transfer to the ubiquitin-conjugating enzyme E2, and recognition of the targeted protein by E3 ubiquitin ligases. Ubiquitin ligase transfers the ubiquitin or polyubiquitin chain from E2 to the lysine residue of the substrate or the N-terminal residue of the protein, which determines the specificity of different ligases regarding the ubiquitination of their substrates (Ciechanover and Ben-Saadon, 2004). Approximately 600–1,000 types of E3 ligases have been identified in the human genome (Li et al., 2018). E3 ligases are divided into the following three major families according to different domains and mechanisms of interaction with substrate proteins: the HECT domain family, the ring finger domain family, and the U-box domain family (Hatakeyama and Nakayama, 2003; Komander and Rape, 2012).

**FIGURE 1:**
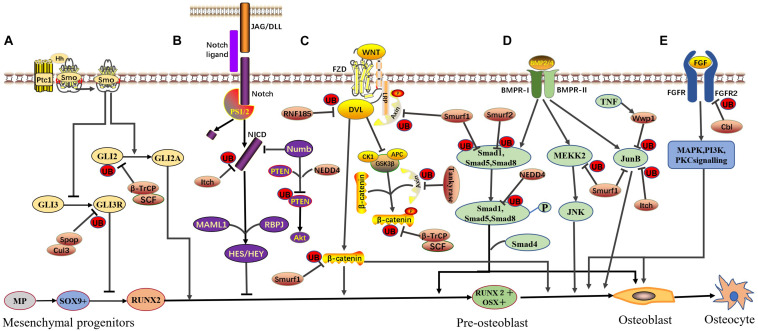
E3 ubiquitin ligase-mediated signaling pathways in osteoblast differentiation. Various E3 ubiquitin ligases and signaling pathways function in a coordinated manner to ensure osteoblast differentiation. The involved signaling pathways are described below. **(A)** Hedgehog signaling: SCF^β–*TrCP*^ directly interacts with phosphorylated Gli2 and promotes Gli2 ubiquitination and degradation, negatively regulating HH signaling. Spop, part of the Cul3 ubiquitin ligase complex, negatively regulates HH signaling by targeting Gli3R for ubiquitination and degradation. **(B)** Notch signaling: Itch binds the N-terminal portion of the NICD and promotes NICD ubiquitination and degradation, negatively regulating Notch signaling. NEDD4 forms a complex with Numb and PTEN, leading to the ubiquitin-mediated proteasomal degradation of PTEN and positively regulating Notch signaling. **(C)** Canonical Wnt signaling: RNF185 targets Dvl2, promotes Dvl2 ubiquitination and degradation, and negatively regulates WNT signaling. Tankyrase stimulates the ubiquitination and degradation of Axin and positively regulates WNT signaling. Smurf1 mediates Lys29-nonproteolytic polyubiquitination degradation of Axin, physically interacts with β-catenin, promotes β-catenin ubiquitination and degradation, and negatively regulates WNT signaling. SCF^β–*TrCP*^ specifically recognizes GSK3β-phosphorylated β-catenin, promotes β-catenin ubiquitination and degradation, and negatively regulates WNT signaling. **(D)** BMP signaling: Smurf1 targets Smad1, MEKK2, and JunB, leading to their ubiquitination and degradation and negatively regulating BMP signaling. Smurf2 can also target Smad1 for ubiquitination similar to, but independent of, Smurf1. WWP1 (under TNF-induced conditions) and Itch can also target JunB and promote JunB ubiquitination and degradation, negatively regulating BMP signaling. **(E)** FGF signaling: Cbl can mediate the ubiquitination and degradation of FGFR2, subsequently inhibiting ERK1/2 and PI3K and negatively regulating FGF signaling. This figure is based on and modeled after Figure 4 in the review by Salhotra et al. (2020).

In the skeletal system, osteoblasts are the main functional cells associated with bone formation, and their proliferation, differentiation, and maturation stages are regulated by various cytokines, transcription factors, and signaling pathways. Studies have shown that E3 ligase plays an important role in maintaining the normal physiological functions of osteoblasts (Sévère et al., 2013; Kim et al., 2020). Notably, inhibition of E3 ubiquitin ligases, such as Smurf1, leads to enhanced osteoblast differentiation, bone formation, and bone mass (Sévère et al., 2013). Thus, E3 ligase may constitute a new therapeutic target for the treatment of osteoporosis. In this review, we summarize current progress in the understanding of the function and regulatory effects of E3 ligases on the key transcription factors and signaling pathways (Figure 1) that regulate osteoblast differentiation and bone formation.

## Transcription Factors Targeted by E3 Ubiquitin Ligases

### Runx2

Runx2 is a member of the Runt domain transcription factor family. It participates in the transduction of multiple signaling pathways and plays an essential role in osteoblast differentiation (Long, 2011). Ubiquitin-proteasome degradation is an important method of the posttranslational regulation of the Runx2 protein (Gomathi et al., 2020; Kim et al., 2020). The HECT domain E3 ligase Smad ubiquitination regulatory factor 1 (Smurf1) was the first identified Runx2 ubiquitination ligase (Zhao et al., 2003), which directly interacts with Runx2 and mediates Runx2 degradation in a ubiquitin- and proteasome-dependent manner, thereby inhibiting osteoblast differentiation and bone formation (Zhao et al., 2004). Upregulation of Smurf1 and Smurf2 expression also mediates tumor necrosis factor (TNF)-induced Runx2 degradation (Kaneki et al., 2006). In contrast, protein kinase B (PKB)/Akt can indirectly enhance the stability and transcriptional activity of Runx2 by regulating ubiquitin-mediated degradation of Smurf2 (Choi et al., 2014). In addition to Smurf1, the HECT class E3 ligase WWP1 can also mediate Runx2 degradation. Schnurri-3 (Shn3), a zinc finger adapter protein, controls the Runx2 protein levels by recruiting WWP1 to Runx2 to promote Runx2 degradation, and consistently, depletion of the Shn3 gene significantly increases the Runx2 protein levels, osteoblast function, and bone mass (Jones et al., 2006). In addition to polyubiquitination, Runx2 is also monoubiquitinated by WWP2, resulting in transactivation of Runx2 (Zhu et al., 2017).

S-phase kinase-associated protein 2 (Skp2), an SCF (SKP1-CUL1-F-box) family E3 ligase that belongs to the ring finger domain family, negatively regulates osteogenesis through the ubiquitin-mediated proteasomal degradation of Runx2, and Skp2 depletion enhances Runx2 expression and osteoblast differentiation (Thacker et al., 2016). The U-box family E3 ligase C-terminus of Hsc70-interacting protein (CHIP) is also involved in Runx2 ubiquitination and degradation. CHIP overexpression in a preosteoblastic cell line can promote Runx2 degradation and inhibit osteoblast differentiation, while knockdown of CHIP can enhance osteoblast differentiation (Li et al., 2008). The study also showed that the level of endogenous CHIP protein gradually decreased as the level of Runx2 protein increased during osteoblast differentiation, but that levels of the E3 ligases Smurf1 and Shn3/WWP1, which are also involved in regulating Runx2, remained constant or increased. A possible explanation is that CHIP, Smurf1, and WWP1 are responsible for Runx2 degradation but function at different stages of osteoblast differentiation, which keeps Runx2 at a low level in pre-osteoblasts, then rising in immature osteoblasts and downregulated again in mature osteoblasts (Komori, 2020). Unfortunately, the ubiquitination sites by these three ligases in Runx2 are currently unknown. Therefore, more research investigating Runx2 ubiquitination in osteoblast differentiation should be conducted.

### Osterix (Osx)

Osterix, an osteoblast transcription factor containing zinc fingers, triggers the differentiation of immature osteoblasts into mature osteoblasts and plays a key role in bone formation (Baek et al., 2009, 2010; Zhou et al., 2010). Peng et al. (2013) identified K58 and K230 as two ubiquitination sites in Osx and revealed that the ubiquitin-proteasome pathway affects the stability and transcriptional activity of Osx and thereby regulates osteoblast differentiation. Another study showed two other ubiquitination sites in Osx (K55 and K386) and indicated that TNF-α promotes Osx degradation in osteoblasts by upregulating the E3 ubiquitin ligase CHIP, thereby inhibiting osteoblast differentiation (Xie and Gu, 2015). The Cbl-b and c-Cbl proteins are members of the mammalian Casitas B-lineage lymphoma (Cbl) family and can function as E3 ubiquitin ligases to participate in the regulation of osteoblast differentiation (Brennan et al., 2011; Sévère et al., 2011; Dieudonne et al., 2013). These proteins have been shown to enhance the ubiquitination and degradation of Osx and then negatively regulate osteoblast differentiation (Choi et al., 2015).

F-box/WD repeat-containing protein 7 (Fbw7) is one of the best-characterized members of the F-box protein family and serves as a receptor subunit of the Skp1-Cullin1 (Cul1)-F-box protein (SCF) E3 ligase complex. Hoshikawa et al. (2020) revealed that p38 and Fbw7 cooperatively target Osx and promote Osx ubiquitination and degradation. Mechanistically, p38-mediated S73/77 Osx phosphorylation promotes the Fbw7 interaction, triggers subsequent Osx ubiquitination, and inhibits osteoblast differentiation.

F-box protein 25 (FBXO25) is also a member of the F-box protein family and contains a ubiquitinated target-binding domain that can induce protein degradation *via* the ubiquitination pathway (Baumann et al., 2014). FBXO25 increases monoubiquitination of histone H2A monoubiquitinated at lysine 120 (H2BK120), promoting trimethylation of histone 3 trimethylated at lysine 4 (H3K4); together, these two modifications induce or enable Osx transcription. When osteogenic differentiation inhibitory lncRNA 1 (ODIR1) expression is high in human umbilical cord-derived mesenchymal stem cells (hUC-MSCs), the E3 ubiquitin ligase Cullin-3 (Cul3) is recruited and promotes the degradation of FBXO25, which leads to downregulation/inhibition of Osx transcription (He et al., 2019).

## Signaling Pathway Regulation

### Hedgehog (HH) Signaling

The Hedgehog signaling pathway is essential for early bone formation. This pathway participates in the regulation of bone morphogenetic proteins (BMPs) and cooperates with BMP2 signaling in osteoblast differentiation and proliferation (Zhao et al., 2006). The Indian Hedgehog (IHH) ligand binds to the cell surface receptor Patched (Ptc) to remove the inhibitory effect of Ptc on Smoothened (Smo). Subsequently, Smo activates the Gli2 activator (Gli2A) and prevents the cleavage of Gli3 into the Gli3 repressor (Gli3R), leading to the expression of SOX9 and Runx2 in osteochondroprogenitor cells (Park et al., 2010; Salhotra et al., 2020).

Studies have shown that proteasome inhibitors can directly act on the Hedgehog signaling pathway and reduce the degradation of the “zinc finger structure” transcription factor Gli2, suggesting that UPS mediates Gli2 degradation (Liu, 2019). Phosphorylated Gli2 directly interacts with β-TrCP in the SCF ubiquitin–ligase complex and promotes β-TrCP ubiquitination and proteasomal degradation, thereby inhibiting BMP2 expression and bone formation (Bhatia et al., 2006), and inhibiting microtubule aggregation can reduce the ubiquitination-mediated degradation of Gli2 by SCF^β–*TrCP*^ and promote BMP2 expression and bone formation (Zhao et al., 2006, 2009).

Speckle-type POZ protein (Spop), which is part of the Cul3 ubiquitin ligase complex, has been shown to negatively regulate HH signaling by targeting Gli2 and Gli3 for ubiquitination and degradation (Wang et al., 2010). However, in contrast to previous studies, Cai and Liu (2016) revealed that Spop plays a positive role in HH signaling. These authors found that chondrocyte and osteoblast differentiation was defective in the absence of Spop. Strikingly, Gli3R and the full-length form of Gli3, but not Gli2, were upregulated in Spop mutants. Consistent with this finding, skeletal defects can be rescued by reducing the Gli3 dosage in Spop mutants, indicating that Spop may regulate IHH signaling specifically by targeting Gli3R for ubiquitination and degradation. Since the expression of mammalian Spop is not limited to cells with active HH signaling (Chen et al., 2009), accumulation of Gli3R in cells with intermediate levels of HH signaling may be the reason for the unique reduction in HH pathway activation in Spop mutant mice (Cai and Liu, 2016).

### Notch Signaling

Notch signaling is a highly conserved cell communication system that plays a negative role in osteoblast differentiation. After binding its ligand (Delta-like ligands, DLL1/3/4; Jagged ligands, JAG1/2), Notch is cleaved to release the Notch intracellular domain (NICD) (Schroeter et al., 1998). In the nucleus, NICD forms a complex with Recombination Signal Binding Protein for Immunoglobulin Kappa J Region (RBPJ) and Mastermind-like protein 1 (MAML1) and then regulates the downstream targets Hairy and Enhancer of Split (HES) and HES related with YRPW motif (HEY), thereby inhibiting osteoblast differentiation (Schroeter et al., 1998; Tu et al., 2012; Zanotti and Canalis, 2016).

Itch, an E3 ligase that belongs to the HECT family, is known to directly target Notch1 and promote ubiquitin-mediated Notch1 degradation (Qiu et al., 2000; Matesic et al., 2006). Itch binds to the N-terminal portion of the NICD through its WW domains and promotes Notch ubiquitination *via* its HECT ubiquitin ligase domain (Qiu et al., 2000). The signals mediated by Notch are increased in Itch-deficient bone marrow mesenchymal stromal/stem cells (BM-MSCs), leading to reduced MSC differentiation into osteoblasts, and therefore resulting in osteopenic bone phenotype (Schunemann et al., 2017). However, another study showed an opposite result with an increased bone volume in Itch-deficient mice (Zhang and Xing, 2013). The reason should be that Itch can also target and promote the protein degradation of JunB, thereby inhibiting the differentiation of bone marrow mesenchymal precursor cells (BM-MPCs) into osteoblasts (Zhang and Xing, 2013).

Numb is a membrane-associated adaptor protein that determines cell fate. Numb inhibits Notch signaling by regulating Notch endocytosis/recycling and NICD ubiquitin/proteasomal degradation (Giebel and Wodarz, 2012). A recent article by Luo et al. (2020) also reported that Numb stabilizes the NICD domain of Notch1 by facilitating the association between NICD and BAP1 (a deubiquitinating enzyme). Nevertheless, Ye et al. (2018) found that the osteopenic phenotype occurred independent of Notch signaling activation in mice with specific ablation of both Numb and its homolog Numbl. According to these authors, Numb maintains bone mass by promoting ubiquitin-mediated degradation of PTEN in osteoblasts. When Numb is expressed normally in osteoblasts, Numb forms a complex with NEDD4 and PTEN, leading to the ubiquitin-mediated proteasomal degradation of PTEN. In contrast, if Numb is suppressed, the accumulation of PTEN in the cytoplasm negatively regulates bone formation by inhibiting the Akt pathway.

### Canonical WNT Signaling

Canonical WNT signaling plays a positive role in osteoblast differentiation. WNT signaling is activated through the binding of WNT ligands to their cell surface receptors, which are low-density lipoprotein receptor-related protein (LRP5/6) and Frizzled (FZD). Upon binding their receptor complex, the cytoplasmic protein disheveled (Dvl) is recruited, phosphorylated, and activated. Dvl activation inhibits the phosphorylation and degradation of β-catenin by the dissociation of GSK-3β from Axin and the subsequent inactivation of the degradation complex. Finally, stabilized β-catenin translocates to the nucleus, leading to changes in the target gene transcriptions, marking the commitment to osteoblast maturation (Baron and Kneissel, 2013; Lerner and Ohlsson, 2015).

The E3 ligase RNF185 contains ring fingers and has been identified as a candidate endogenous suppressor of osteogenic specification in human MSCs (hMSCs) (Zhao and Ding, 2007). RNF185 overexpression reduces the level of Dvl2 by promoting Dvl2 ubiquitination and degradation and inhibits β-catenin-mediated transcriptional activity, thereby inhibiting osteoblast differentiation. Dvl2 reverses the inhibitory effect of RNF185 on the osteogenic differentiation of MC3T3-E1 cells (Zhou et al., 2014). These results indicate that Dvl2 is a ubiquitinated substrate of RNF185. Interestingly, a recent study using HEK293 cells showed that Dvl can regulate the activity of Smurf2, which allows Smurf2 to more effectively ubiquitinate substrates of the WNT/PCP (Prickle1) and TGF-β/BMP (Smad2) pathways (Bernatik et al., 2020). Therefore, Dvl may act as a point of cross talk between the WNTt and TGF-β/BMP pathways.

The β-catenin destruction complex is a target of several ubiquitin ligases, and the Axin protein is the main substrate of UPS-dependent regulation (Ji et al., 2019). Tankyrase is a poly (ADP-ribose) polymerase that can stimulate the ubiquitination and degradation of Axin and subsequently upregulate WNT/β-catenin signaling (Huang et al., 2009). However, the pharmacological inhibition of tankyrase enhances osteoblast differentiation and maturation, suggesting that other molecules are involved in promoting osteoblastogenesis, despite the inhibition of WNT/β-catenin signaling (Fujita et al., 2018). A possible explanation is that another substrate of tankyrase, the adaptor protein SH3 domain-binding protein 2 (SH3BP3) (unrelated to canonical WNT signaling), accumulates during the administration of tankyrase inhibitors, which, in turn, positively regulates osteoblastogenesis (Fujita et al., 2018; Mukai et al., 2019). Most importantly, the systemic administration of a tankyrase inhibitor resulted in bone loss with increased numbers of osteoclasts (Fujita et al., 2018; Mukai et al., 2019).

As a C2-WW-HECT-type E3 ubiquitin ligase, Smurf1 is largely known for its role in several signaling pathways. Smurf1 negatively regulates WNT/β-catenin signal transduction by mediating Lys29-nonproteolytic polyubiquitination of Axin (Fei et al., 2013). Smurf1 disrupts the interaction between Axin and LRP5/6, which subsequently attenuates WNT-stimulated LRP6 phosphorylation and inhibits WNT/β-catenin signaling (Fei et al., 2013). In addition, Smurf1 is a biologically relevant E3 ligase that promotes the ubiquitination and degradation of β-catenin, although the PPXY motif is absent from β-catenin (David et al., 2013; Boonanantanasarn et al., 2015). In some cell lines other than osteoblasts, Axin can also be ubiquitinated by other E3 ubiquitin ligases. Kim and Jho (2010) found that the transient expression of Smurf2 downregulated the level of Axin through its ubiquitin-mediated proteasomal degradation of Axin and led to a decrease in the activity of the β-catenin/Tcf reporter. RNF146, a poly (ADP-ribose)-directed E3 ligase, is also an E3 ligase responsible for Axin degradation and promotes WNT signaling by mediating tankyrase-dependent degradation of Axin (Callow et al., 2011; Zhang et al., 2011).

The RING-like E3 ligase SCF^βTrCP^ participates in the regulation of the WNT signaling pathway and mediates the degradation of β-catenin *via* the ubiquitin/proteasome pathway, thereby functioning as an important regulator of osteoblast differentiation (Ougolkov et al., 2004). Without the WNT ligand, GSK3β-phosphorylated β-catenin is recognized by the F-box-containing ubiquitin E3 ligase β-TrCP, leading to β-catenin ubiquitination and rapid degradation by the 26S proteasome (Jiang and Struhl, 1998). BMP-2 and β-catenin can synergistically promote osteoblast differentiation. Studies have shown that BMP-2 can promote the gene expression of LRP5 and inhibit expression of the E3 ligase SCF^βTrCP^, which specifically recognize β-catenin, reduce β-catenin ubiquitination, and promote the proliferation and differentiation of osteoblasts (Zhang et al., 2009).

### BMP Signaling

The BMP signaling pathway plays an important biological role in bone development and bone formation after birth and is involved in regulating the differentiation and function of MSCs, osteoblast precursor cells, and mature osteoblasts (Beederman et al., 2013). The Smad-dependent BMP pathway initiates signal transduction by binding to cell surface receptors and then phosphorylates the intracellular transducers Smad1/5/8. Phosphorylated Smad1/5/8 (pSmad1/5/8) interacts with Smad4 and translocates into the nucleus, where these proteins interact with Runx2 to induce osteogenic gene expression (Sapkota et al., 2007; Beederman et al., 2013). Non-Smad-dependent pathways include the extracellular signal-regulated kinase (ERK), c-Jun amino-terminal kinase (JNK), and p38 MAP kinase (MAPK) pathways. BMP-2 can also activate JNK and p38 *via* the protein kinase D (PKD) pathway to promote osteoblast differentiation. Therefore, BMP stimulates both the Smad and p38 MAPK pathways and converges at Runx2 to regulate the differentiation of MSCs (Lee et al., 2002).

Smads are indispensable and important components of BMP signaling pathways, and the interaction between the E3 ligase Smurf1 and Smad1 participates in the regulation of bone formation. Studies have shown that Smurf l mediates the degradation of Smad1, a downstream factor regulated by BMP receptors, thereby inhibiting BMP-induced osteoblast differentiation (Zhu et al., 1999; Zhao et al., 2004). Molecular biology studies have shown that Smurf1 specifically recognizes the PY motif of Runx2 and Smad1 through its WW domain and then mediates the polyubiquitination and degradation of Runx2 and Smad1 through the 26S proteasome, which negatively regulates bone formation through the BMP signaling pathway (Xing et al., 2010). Smurf1-knockout mice exhibited increased osteoblast activity at 4 months of age, which, in turn, resulted in an increased bone mass. However, there was no increase in BMP signaling or Runx2 activity, while the JNK signaling pathway was continuously activated (Yamashita et al., 2005). In preosteoblasts or mature osteoblasts, Smurf1 can target the upstream activator of JNK, MAPK/ERK kinase kinase 2 (MEKK2), and mediate its ubiquitination degradation, indicating that MEKK2 is an important substrate protein of Smurf1 in osteoblasts (Yamashita et al., 2005; Sun et al., 2018). Additionally, Smurf1 can specifically recognize and interact with the JunB PY motif, thereby mediating the ubiquitination of JunB and negatively regulating the proliferation and differentiation of MSCs (Zhao et al., 2010). Therefore, Smurf1 influences BMP signaling by targeting components of Smad-dependent and Smad-independent downstream branches.

In addition to Smurf1, Smurf2 has been shown to negatively regulate BMP/Smad signaling *via* ubiquitination of Smad3 (Kushioka et al., 2020). In *Xenopus* embryos, Smurf2 also targets Smad1 for ubiquitination, similar to, but independent of, Smurf1 (Zhang et al., 2001). The role of Smurf2 in osteoblasts is similar to that observed in Smurf1 loss-of-function studies in terms of increased osteoblast differentiation (Yamashita et al., 2005). In contrast to the increased bone mass phenotype of Smurf1^–/–^ mice, Smurf2^–/–^ mice exhibit reduced bone mass and increased bone resorption (Xu et al., 2017). Mechanistically, Smurf2 upregulates the expression of RANKL by disrupting the interaction between Smad3 and the vitamin D receptor by altering Smad3 ubiquitination, thereby increasing the number of osteoclasts and bone resorption (Xu et al., 2017).

Neural precursor cells express developmentally downregulated protein 4 (NEDD4), another E3 ubiquitin ligase belonging to the HECT family. NEDD4 promotes osteoblast proliferation by degrading pSmad1 activated *via* TGFβ1 and potentiating the pSmad2 and pERK1/2 pathways in the early stage of bone formation (Jeon et al., 2018). The HECT-like E3 ligase members WWP1 and Itch also target JunB to participate in osteogenesis regulation. WWP1 mediates ubiquitination and degradation of JunB, but TNF-induced conditions are necessary (Zhao et al., 2011). Related studies have shown that the osteogenic differentiation of MSCs in TNF transgenic mice is significantly reduced, while the expression of WWP1 is increased. Inhibiting the expression of WWP1 can reverse the osteogenic differentiation of MSCs in TNF-Tg mice (Zhao et al., 2011). The E3 ligase Itch can also target and promote the protein degradation of JunB and inhibit osteoblast differentiation. The bone volume and bone formation rate in Itch-knockout mice are significantly increased (Zhang and Xing, 2013). Arkadia, a RING-type E3 ubiquitin ligase, positively regulates BMP signaling *via* the degradation of Smad6, Smad7 and c-Ski/SnoN, thereby promoting the differentiation of primary osteoblasts (Tsubakihara et al., 2015).

### Fibroblast Growth Factor (FGF) Signaling

Fibroblast growth factors bind one of four transmembrane receptors with intracellular tyrosine kinase domains (FGFR1–FGFR4), leading to the cross-phosphorylation of tyrosine residues in the intracellular domain of receptor tyrosine kinase (RTK). These phosphorylated residues are specifically bound by several intracellular signal transduction proteins to activate several intracellular signaling pathways, including the Ras-MAPK, phosphoinositide 3-kinase-AKT, Jak-STAT, and protein kinase C pathways (Ornitz, 2005). FGF signaling regulates embryonic development and various cellular physiological activities, especially in osteogenesis, which controls preosteoblast proliferation, osteoblast differentiation, and the function of mature osteoblasts (Ornitz and Marie, 2015).

As previously described, the E3 ubiquitin ligase Cbl negatively regulates osteoblast differentiation (Choi et al., 2015). Accumulation of Cbl at the RTK site and induced polyubiquitination and degradation of multiple RTKs are important regulatory mechanisms of osteoblast function (Sévère et al., 2013). Cbl can mediate the ubiquitination and degradation of platelet-derived growth factor receptor (PDGFR) and fibroblast growth factor receptor 2 (FGFR2), thereby inhibiting the directed osteogenic differentiation of hMSCs. Specific inhibition of Cbl and RTK interaction using the Cbl mutant (G306E) resulted in increased expression of PDGFRα and FGFR2 in hMSCs, which activated the ERK1/2 and PI3K signaling pathways and promoted the expression of osteoblast markers and osteogenic differentiation of MSCs. As the inhibitory effects of drugs on FGFR2 or PDGFR counteract the *in vitro* osteogenesis induced by the Cbl mutant (Sévère et al., 2011), Cbl primarily suppresses osteoblast differentiation by targeting Osx and FGFR2 *via* ubiquitination for degradation (Sévère et al., 2011).

The stability of Smurf1 can also be regulated by other E3 ligases, such as casein kinase 2-interacting protein-1 (CKIP-1, also known as PLEKHO1) and Cdh1. Studies have shown that Cdh1 enhances the activity of Smurf1 by reducing the self-inhibition of the Smurf1 homodimer and that Cdh1 gene knockdown reduces the activity of Smurf1 and its downstream signaling pathways (including the MEKK 2 signaling pathway), thereby affecting osteoblast differentiation (Wan et al., 2011). CKIP-1 was previously identified as a ubiquitination-related molecule that could specifically target the linker region between the WW domains of Smurf1 to promote the ubiquitination of Smad1/5 (Lu et al., 2008). It has been speculated that CKIP-1 functions to increase Smurf1 affinity by stabilizing WW domain coupling (Chong et al., 2010). Inhibiting CKIP-1 gene expression in osteoporosis model rats enhances osteoblast differentiation and bone formation (Zhang et al., 2012), whereas increasing CKIP-1 expression can suppress Smad-dependent BMP signaling to inhibit bone formation (Liu et al., 2017a,b). In addition, the cullin E3 ligase complex named SCF^FBXL15^ ubiquitinates Smurf1 and induces its proteasomal degradation (Cui et al., 2011).

## Clinical Applications and Prospects

Relevant clinical studies have shown that the proteasome inhibitor bortezomib can promote osteogenesis and inhibit bone resorption in patients with multiple myeloma (Accardi et al., 2015). Mechanistically, bortezomib can promote bone formation by reducing the proteasomal degradation of Runx2 to promote the osteogenic differentiation of MSCs (Mukherjee et al., 2008), and this drug can reduce the expression of the extracellular WNT/β-catenin signaling pathway antagonist Dickkopfl (DKK1) (Oyajobi et al., 2007). The proteasome inhibitor lactacystin can also enhance BMP-induced osteoblastic differentiation by increasing active Smad levels (Ito et al., 2011). Melatonin is a highly evolutionarily conserved molecule with multiple biological functions. Studies have shown that melatonin treatment can downregulate the TNFα-induced expression of Smurf1 and reduce the Smurf1-mediated ubiquitination and degradation of Smad1, resulting in stable BMP/Smad1 signaling activity and the restoration of TNFα-impaired osteogenesis (Lian et al., 2016). A chalcone derivative, which was identified as a small molecular inhibitor targeting Smurf1, can interact with the WW1/2 domains of Smurf1, effectively inhibit its activity, and enhance BMP signaling. In BMP-2^n^/Smurf1^e^ (normal BMP-2 levels and elevated Smurf1 activity) mice, the chalcone derivative enhances local bone formation during spinal fusion (Liang et al., 2018). In addition, some researchers developed the specific DNA aptamer C3A, which can bind WWP1 and inhibit WWP1 ubiquitination of Runx2, thereby increasing the deposition of the extracellular matrix (Tucker et al., 2018). Recent studies have shown that compounds or natural products, such as catalpol (Meng et al., 2020), carnosic acid (Zheng et al., 2020), norlichexanthone (Wang et al., 2021), and RTA-408 (Sun et al., 2020), can suppress RANKL-induced osteoclast formation by regulating factor or receptor ubiquitination and proteasomal degradation, thereby inhibiting bone loss. These findings all indicate that ubiquitination-targeted therapy is a promising strategy for the treatment of osteoporosis.

The ubiquitination–proteasome and degradation system is an essential process that regulates the homeostasis and function of intracellular proteins. As a link between ubiquitin and substrate proteins, the ubiquitin ligase E3 specifically recognizes a substrate and modulates the tyrosine kinase receptors, signal proteins, and transcription factors involved in the regulation of osteoblast proliferation, differentiation, survival, and bone formation. Overall, increased understanding of the function of the ubiquitin ligase E3 in osteoblast differentiation and bone formation provides a rationale for developing E3-targeted therapeutics for the treatment of osteoporosis. However, as there are many E3 ligase substrate proteins, the clinical application of E3 ligase inhibitors may not only affect the skeletal system. Therefore, more comprehensive and systematic research investigating ubiquitination and its regulation in the skeletal system should be conducted.

## Author Contributions

JS, BF, and HuL reviewed the literature and wrote the manuscript. YL, YW, and HZ prepared the literatures and figure. HS, HaL, and WH planned the study and revised the manuscript. All the authors gave the final approval of the manuscript.

## Conflict of Interest

The authors declare that the research was conducted in the absence of any commercial or financial relationships that could be construed as a potential conflict of interest.

## Publisher’s Note

All claims expressed in this article are solely those of the authors and do not necessarily represent those of their affiliated organizations, or those of the publisher, the editors and the reviewers. Any product that may be evaluated in this article, or claim that may be made by its manufacturer, is not guaranteed or endorsed by the publisher.
